# A systematic review of telemedicine for stroke in South-East Asia Region

**DOI:** 10.1186/s12883-026-05204-w

**Published:** 2026-08-03

**Authors:** Lisda Amalia, Abdurahman Harits, Deidre Anne De Silva

**Affiliations:** 1 Department of Neurology, Medical Faculty, Universitas Padjadjaran, Jl.Eykman 38, Bandung, 40161 Indonesia; 2 Department of Neurology, National Neuroscience Institute (Singapore General Hospital Campus), Singapore, Singapore

**Keywords:** Acute stroke, Telestroke, Clinical outcome, SEAR

## Abstract

Telestroke is an innovation in telemedicine in the form of a method for identifying, assessing, treating and monitoring stroke patients remotely using internet technology. Telestroke can speed up the diagnosis and treatment of acute stroke through direct video consultations between doctors and patients separated by distance. The aim of this systematic review is to analyze the implementation of telestroke in improving the clinical outcomes of acute stroke patients in the South-East Asia Region (SEAR). The literature search was carried out through 2 electronic databases, namely PubMed and ScienceDirect which include articles published up to 31 December 2024, and published in English. Before selecting the studies, the study duplication was detected by Zotero Reference Manager^®^ and then removed. The related studies examined and selected with rayyan.ai^®^ website using a “blind-on” mode to ensure eligibility. A total of 6 studies were included. We found that there were 3 types of telestroke programs being implemented, namely remote radiology teleconsultation, drip and ship methods, and also international teleconsultation between Thailand and Laos. Telestroke can provide better opportunities and associated with the clinical outcomes of acute stroke patients with faster door-to-treatment and better 3-month outcomes as well, although published data is still limited. It is hoped that telestroke can bridge this gap, allowing hospitals in rural areas to provide care equivalent to hospitals that have stroke specialists especially in SEAR for improving acute stroke services.

## Introduction

The South-East Asia Region (SEAR), as defined by the World Health Organisation (WHO), has a quarter of the world’s population and covers about 3% of the Earth’s total land area [1]. The South-East Asia Region (SEAR) currently faces a critical epidemiological crisis, accounting for nearly half of the global stroke burden within developing nations and emerging as the leading contributor to stroke-related mortality worldwide [2]. Despite the profound disease burden, emergency medical services and acute stroke management in this region remain profoundly suboptimal. This systemic deficit is evidenced by exceptionally low utilization rates of gold-standard, time-sensitive interventions with low rates of treatments like thrombolysis, mechanical thrombectomy, and Stroke Units (SU) care [3].

Patients in rural areas of SEAR are less likely to get intravenous thrombolysis compared to those in cities [4, 5]. This is because new treatments and technologies aren’t spreading fast enough to rural hospitals, and there’s a lack of access to stroke care and specialists [4]. Moving patients between hospitals to get specialised stroke care also causes delays, which affects how well treatments like thrombolysis and mechanical thrombectomy work [5].

Telestroke is a new medical technology that helps identify, assess, treat, and monitor stroke patients from a distance [6, 7]. Studies show that telestroke can safely and effectively help more patients get thrombolysis, reduce the time between arriving at the hospital and getting treatment, and improve patient outcomes [6, 8]. This review aims to describe the current state of telestroke services for treating strokes quickly in SEAR, giving a background to understand how useful this approach could be for the region.

## Methods

### Research design

A systematic review method was used, following the guidelines from the Joanna Briggs Institute (JBI) [8], and the results were reported using the PRISMA 2020 framework. A literature search was conducted in two electronic databases, PubMed and ScienceDirect, and included articles published up to 31 December 2024. The study focused on two main ideas: telestroke and the South-East Asia Region (SEAR). The search terms included “telestroke,” “telemedicine,” and “teleconsultation,” as well as “SEAR” and the 10 countries in the region: Bangladesh, Bhutan, Democratic People’s Republic of Korea, India, Myanmar, Laos, Nepal, Sri Lanka, Thailand, and Timor-Leste. The terms from each concept were combined using “or,” and then connected with “and.”

The selection of studies began with a review of titles and abstracts. Articles that were relevant were then checked in full by two reviewers, LA and AH, independently. The inclusion criteria were that the article discussed telestroke in the SEAR region and was written in English. Abstracts, conference proceedings (grey literature), clinical trial registries, and short communications were excluded. Duplicates were identified and removed using the Zotero Reference Manager^®^.

The selected studies were reviewed on the rayyan.ai^®^ website using a “blind-on” mode to ensure fairness. If there was any disagreement between the two reviewers, the third reviewer, DADS, provided their opinion to resolve the issue. Data from the included studies were collected into a spreadsheet. The spreadsheet included details like the authors’ names, the year of publication, the participants or population studied, the type of telestroke, and the outcomes. The outcomes included intravenous thrombolysis (IVT) rate, time from symptom onset-to-treatment (OTT), 3-month functional outcomes measured by the modified Rankin scale (mRS), symptomatic intracranial haemorrhage (sICH), and any other outcomes that were reported. This study has been registered to PROSPERO with registration number 1,249,815.

We use Cochrane’s risk of bias assessment to evaluates potential systematic errors in trials to ensure reliable conclusions in systematic reviews with the Revised Cochrane risk-of-bias tool for randomized trials (RoB 2).

### Study selection

**Fig. 1:**
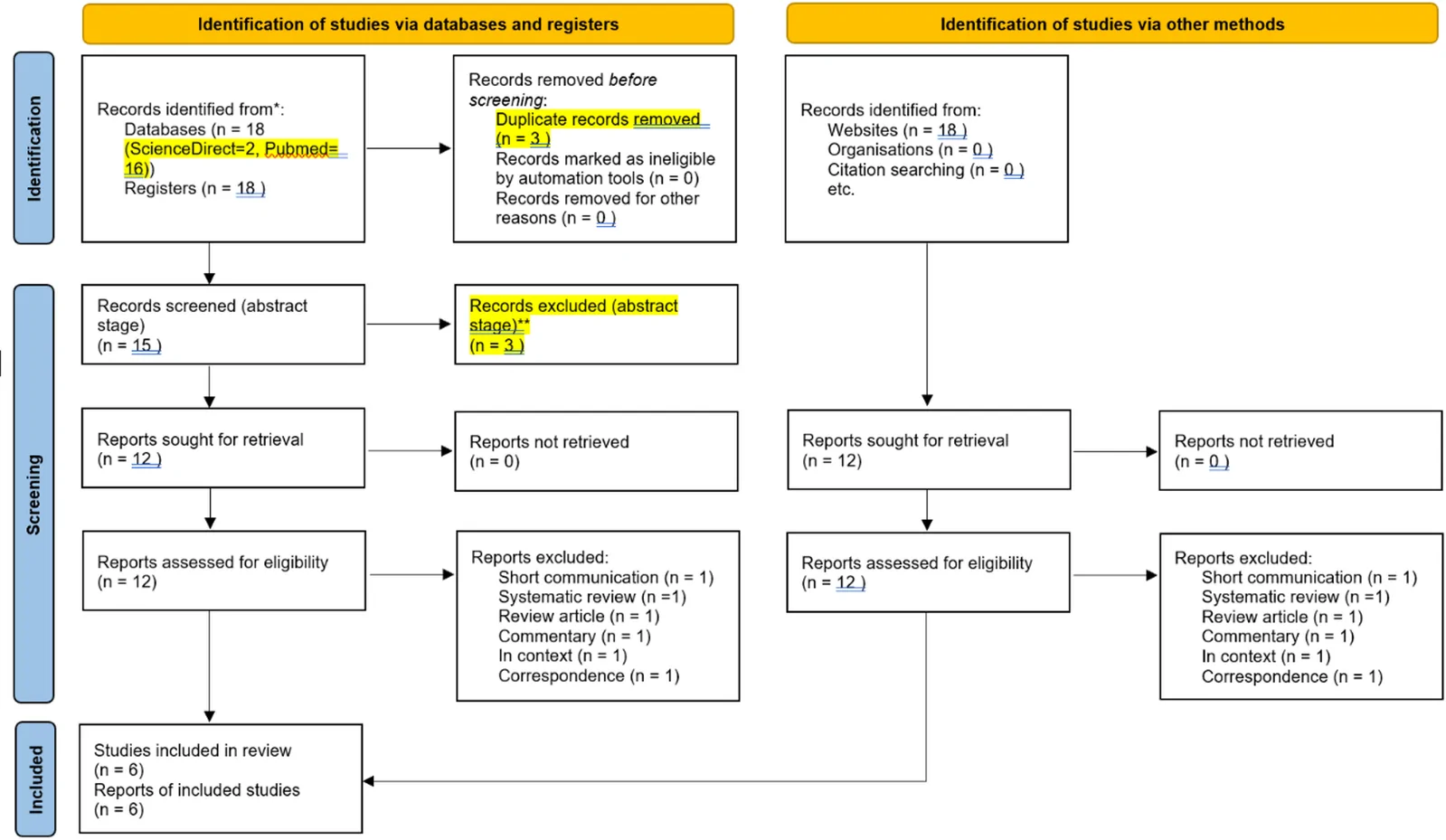
The study selection flow adapted from PRISMA 2020 Flow Diagram For New Systematic Reviews.(Haddaway et al., 2022)

**Fig. 2:**
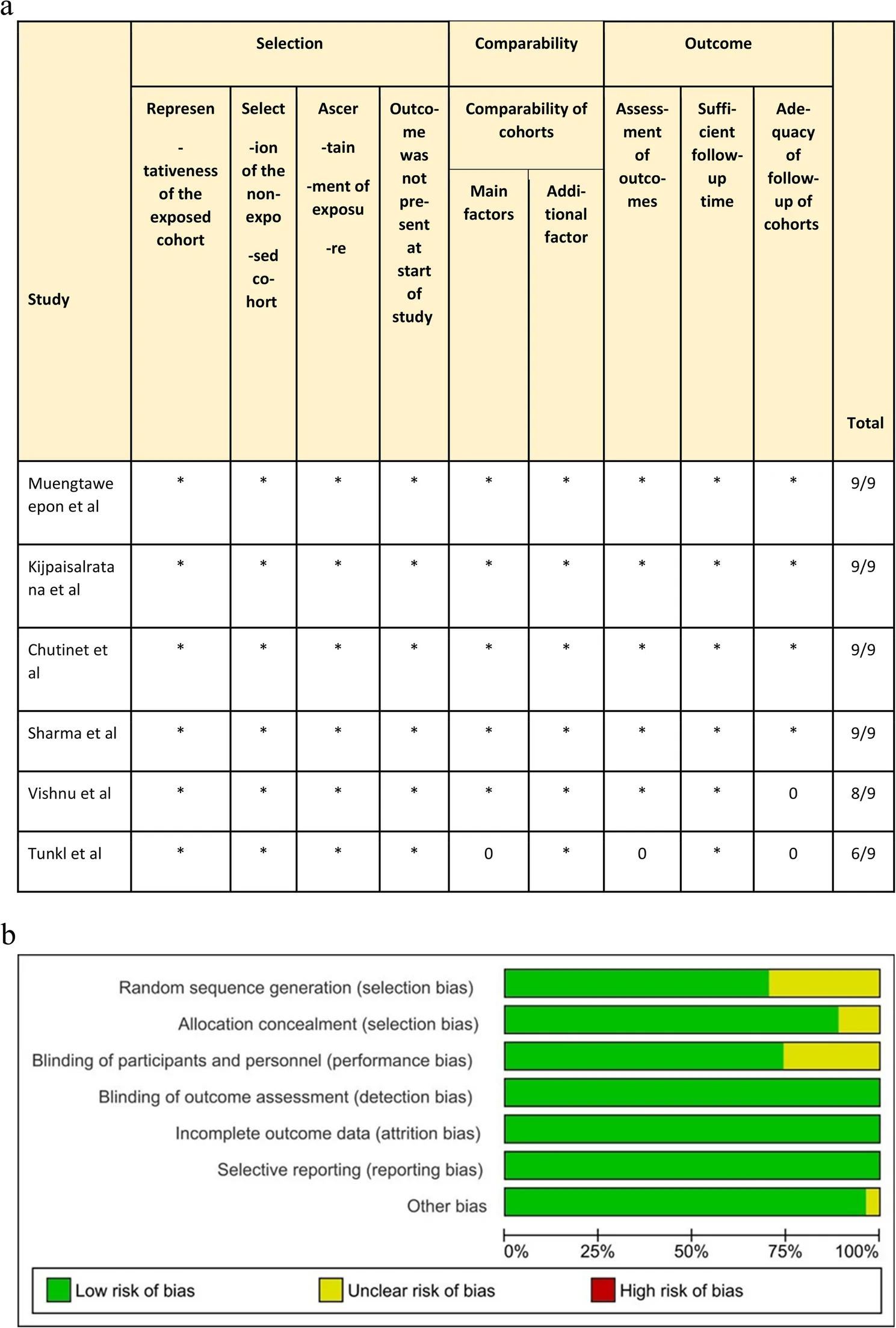
Quality Assessment of the Studies. **a** Risk-of-bias summary: review authors’ judgments about each risk-of-bias item for each included study. **b** Risk-of-bias graph: review authors’ judgments about each risk-of-bias item presented as percentages across all included studies

The search from PubMed and ScienceDirect revealed 18 records. After removal of duplication and screening of title and abstract, six studies were selected. Following full-text review, six studies were included (Fig. 1) and quality assessment of studies (Fig. 2).

## Results

### Study characteristics

Of the six studies included, two were from Thailand, two from India, one from Laos, and one was from a global network. (Table 1).

**Table 1:** Implementation of telestroke in SEAR region

Author	Short-term						
	Year	Countries	Type of telestroke	Number of participant	Technology	Financing	Clinical outcome
Sombat Muengtaweepon et al. (Muengtaweepongsa, 2010) [9]	2010	Thailand	Remote radiology interpretation with teleconsultation	458	Real time teleconsultation	Government Hospital	Timeliness of intravenous thrombolysis (DTN and OTT)
Naruchorn Kijpaisalratana et. al.(Kijpaisalratana et al., 2020) [10]	2020	Thailand	Teleconsultation	273	Teleconsultation from PSC to CSC	Government	Timeliness of intravenous thrombolysis (DTN and OTT)
Aurauma Chutinet et al. (Chutinet et al., 2019) [11]	2019	Laos	International teleconsultation with Thailand	28	Real time teleconsultation	Government hospital	Facilitate the thrombolytic therapy for acute ischemic stroke patients in Laos
Sudhir Sharma et al. (Sharma et al., 2016) [12]	2016	India	Teleconsultation	26	Teleconsultation from PSC to CSC	Government hospital	Facilitate the thrombolytic therapy for acute ischemic stroke patients in rural area
Venugopalan Y Vishnu et al. (Vishnu et al., 2022) [13]	2022	India	Teleconsultation	22	Teleconsultation from PSC to CSC (stroke physician to stroke neurologist and physiotherapist)	Government hospital	Facilitate stroke care during acute until chronic phase and improving stroke outcome
Christine Tunkl et al. (Tunkl et al., 2024) [14]	2024	Global network (67 countries)	Telestroke network and teleconsultation	254	Telestroke network using hub-and-spoke teleconsultation (video examination and imaging transfer)	HIC and LIC countries	Facilitate the thrombolytic therapy for acute ischemic stroke patients and improving stroke outcome

Abbreviation: *SEAR *South East Asia Region, *PSC *Primary Stroke Centre, *CSC *Comprehensive Stroke Centre, *DTN *Door-to-needle time, *OTT* Onset-to-needle time, *HIC* High Income countries, *LIC * Low Income countries

One of the studies from Thailand [9], included 458 patients admitted with acute ischemic stroke (AIS) and transient ischemic attack (TIA), of which 100 (21%) patients received intravenous rt-PA with mean door-to-needle (DTN) was 54 min and onset-to-treatment (OTT) was 160 min. There was remote radiology interpretation and telephone consultation for decision-making of intravenous thrombolysis. There were 13 (2%) cases with asymptomatic intracerebral hemorrhages and among these patients (two (15%) cases), one patient died (modified Rankin Scale (mRS) = 6) and the other had poor functional outcome (mRS = 5). At 3-months, 42% patients had achieved excellent recovery mRS was 0–1. The conclusions of this report were that the telestroke systems was feasible and safe, and was well received. The authors opined that further studies are needed to determine the benefit of this method as compared to the conventional telephone consultation alone.

The other study from Thailand (Kijpaisalratana et al., 2020) included 273 participants using telestroke consultation was then initiated with real-time interactive videoconferencing. DTN and OTT duration (63.0 ± 44/166.09 ± 87 min) was significantly longer in ship-and-drip group compared to mothership and drip-and-ship. For clinical and safety outcomes of patients receiving thrombolytic treatment, there was no statistical significance in functional independence at 3 months among patients receiving thrombolytic treatment in this three different mode. Telestroke facilitates acute stroke care by connecting onsite physicians with stroke experts to help increase use of thrombolytic treatment and improve the number of acute ischemic stroke patients receiving effective treatment with three different mode of rtPA delivery including mothership, drip-and-ship, and ship-and-drip paradigms.

The study from Laos [11] described teleconsultation with King Chulangkorn Memoriam Hospital in Thailand. Among 205 patients with acute stroke during the study period of 6 months, 28 of whom (14%) arrived at the hospital within 4.5 h of onset. There were ten patients (5%) were eligible and treated of intravenous thrombolysis. The mean (± SD) time from stroke onset to hospital arrival was 122 (± 74) minutes. The mean (SD) time from stroke onset to thrombolytic treatment was 225 (± 39) minutes. The average door-to-needle time was 108 min; the fastest was 55 min and the longest was 185 min. Three patients (30%) received thrombolysis with the door-to-needle (DTN) time less than 60 min. The mean (± SD) door to CT and door to teleconsultation was 38 (± 23) minutes and 68.8 (± 35) minutes, respectively. Provides evidence of cross-country telestroke possibilities.

There were three reports on one study from India [12–14] with a total of 3562 patients, reported that telestroke with teleconsultation method and telestroke network from Primary Stroke Centre (PSC) to Comprehensive Stroke Centre (CSC) helps improve intravenous thrombolysis procedures for hyperacute ischemic stroke patients in rural areas without neurologist. Smartphone‑based and SMART application experience of telemedicine in acute stroke care in India. As the objective was to demonstrate its feasibility, there was less emphasis on data collection regarding stroke performance indicators such as onset-to-needle time (< 180 min, and door‑to‑needle time (< 60 min).

Previous study [14], the global survey of telestroke networks reveals significant disparities in coverage and implementation, with a clear bias toward High Income Countries (HICs). Telestroke network implementation in HICs is more widely used than Low Income Countries (LICs) by the number of teleconsultations and the number of hospitals involved in the telestroke network, indicating inadequate access to stroke care in Low Middle Income Countries (LMICs). HICs use more high-cost specialized technologies, while other regions rely more on messaging applications. The implementation of telestroke through teleconsultation via the telestroke network plays an important role in improving the clinical outcomes of acute stroke patients because they can receive effective therapy more quickly. More work is required in LMICs to implement telestroke services and support access to stroke reperfusion therapies. The implementation of telestroke in SEAR (some of which is included in LICs) is still not widely used due to constraints in existing technology and policies, but it is still promising because it is very helpful and associated with clinical outcomes of acute stroke patients.

The authors are aware of various other telestroke programmes in SEAR, however there are no published data on these.

### Risk of bias

With the Revised Cochrane risk-of-bias tool for randomized trials (RoB 2), the overall risk of bias was low in six studies, whereas one study had some concerns. Overall, the methodological quality of the included studies was considered acceptable. Figure 2 summarizes the Revised Cochrane risk-of-bias tool for randomized trials (RoB 2) results of the studies.

## Discussion

There are not many research papers about telestroke in this area, even though it has a large population and a high number of stroke cases. This is probably because there are not enough telestroke services available and not enough studies being published about the ones that do exist. In the Southeast Asian Region (SEAR), there is a lot of potential for telestroke services due to its wide area, the concentration of advanced medical services and skilled professionals in urban areas, and the widespread use of phones and the internet. However, there is a big gap between the availability of stroke care and the high number of stroke cases in the region. Most countries in SEAR are classified as low- or middle-income by the World Bank, which means they often lack both proper healthcare facilities and trained medical staff [15]. These factors, along with aging populations, different geographical areas, and various cultural and social backgrounds, make it hard to provide consistent stroke care across the region [6, 16]. Telestroke uses telemedicine tools to help with remote stroke diagnosis, consultation, and treatment, especially in places where specialized care is not easily available [12, 17, 18]. It is especially helpful in areas where there are not enough neurologists or where stroke expertise is limited in rural or underserved areas [7, 15, 19]. In SEAR, telestroke programs are starting to develop, which can help reduce healthcare inequalities and improve results for stroke patients [20]. Stroke care in SEAR countries needs to be improved and made stronger, but it is important to understand the different geographical, cultural, social, and economic differences in these countries [18, 21]. Using telestroke to increase access to stroke care in rural areas is a promising solution. Telestroke networks, digital tools, and mobile apps can help share knowledge within and between countries [14, 16, 22, 23]. Studies have shown opportunities for global health through collaboration between countries, such as between Thailand and Laos, where more developed nations can support others with less developed stroke services and there is potential for international cooperation [22, 24]. Telestroke helps with acute stroke care by linking local doctors with stroke specialists, which can increase the use of clot-busting treatments and associated with outcomes for patients with acute ischemic strokes [16, 17, 19, 20, 25, 26] It can also speed up the diagnosis and treatment of stroke through direct video calls between doctors and patients who are far apart [7, 16]. Different techniques of telestroke in the South-East Asia Region (SEAR), such as teleconsultation or drip-and-ship, enabling them to better navigate their local environments. The drip-and-ship treatment paradigm has contributed to an increase in the rate of thrombolytic treatment in a nationwide study [11, 26–28]. Given the diversity of spoke hospitals, stroke networks have developed various thrombolytic delivery paradigms tailored to each hospital’s capabilities [13, 23, 29]. A study in Thailand demonstrated that the mothership and ship-and-drip protocols showed significantly better outcomes in terms of OTN and DTN compared to the ship-and-drip protocol alone. However, more research is needed on the effectiveness of telestroke implementation in SEAR due to limited sample sizes, although there is supporting evidence from the literature [11, 14]. It is hoped that telestroke can bridge this gap, enabling rural hospitals to provide care comparable to those with stroke specialists [7]. These six published papers illustrate how telestroke can serve as a viable solution. Telestroke can accelerate the diagnosis and treatment of acute stroke through direct video consultations between doctors and patients in different locations [6]. Despite this, there is limited published data, leading to an incomplete understanding of the current utilization of telestroke. Therefore, there is a need for a survey to assess the current state of telestroke in SEAR. To address challenges such as infrastructure limitations, efforts should focus on expanding internet access, enhancing public awareness of stroke symptoms and the importance of timely treatment, and developing supportive policies for telemedicine [4, 10, 29]. Interestingly, although telestroke networks face various operational challenges, particularly technical ones, they are generally viewed as effective tools for improving stroke care by providing access to expert advice and enhancing care quality [26].

Stroke care services in SEAR countries require urgent strengthening and improvement. Multidisciplinary approach should account geographical, cultural/social/economic differences. Setting realistic targets and achieving context-appropriate goals is part of the Global Stroke Action Plan, which recognizes that different systems may be involved in stroke care across the world.To ensure effective primary prevention and reduce treatment delays, it is essential to raise awareness about stroke and risk factors. Behavioral modification programs for stroke awareness and government policies, such as taxes, are necessary to control lifestyle determinants. Countries lacking skilled workers should adopt hub-and spoke, task-sharing, and physician-led models of stroke care as effective approaches. The use of telestroke networks, digital technology, and mobile apps for knowledge sharing can occur both within and between countries. Achieving equitable stroke care in SEAR requires the implementation of evidence-based policymaking, which involves a multidisciplinary approach with active participation from stakeholders such as stakeholders (e.g. healthcare policymakers for telestroke financing program), government agencies, and ministries of health. Enhancing social literacy on stroke through the wide dissemination of stroke awareness programs, specialized stroke recognition training for paramedical staff, establishment of a national stroke hotline number to expedite referrals, and the adoption of hospital prenotification is necessary in order to improve telestroke healthcare services. Further research is needed on the effectiveness and implementation of telestroke in SEAR [26]. It is hoped that telestroke can bridge the gap, allowing rural hospitals to offer care equivalent to those with stroke specialists, especially in SEAR [13, 27, 30].

However, there are several limitations in this study. The authors are aware of other telestroke services through personal communication, but these efforts and their impact have not been documented in published literature. Small number of included studies, differences in study design, lack of meta-analysis, English language restriction, limited database coverage, possible publication bias, and limited comparative clinical outcome data. Given the limited inclusion of English-language studies, we still believe that the role of other non-English-language publications can still be considered. A comprehensive review is needed. Another limitation of study, this systematic review does not discuss the role of government to address challenges such as infrastructure limitations, and economic approaches in assessing the effectiveness of telestroke, so further research is expected to be able to assess it.

## Conclusion

Telestroke can provide better opportunities in SEAR for improving acute stroke services. Telestroke can change how stroke care is provided by connecting urban hospitals that have experts with rural areas that do not have much healthcare support. It can also help and associated with patient results. To make telestroke work well and spread across the region, it is important to keep investing, educate people, and work together at a regional level.

## Data Availability

The datasets generated during and/or analyzed during the current study are available from the corresponding author on reasonable request.

## References

[1] Venketasubramanian N, Yoon BW, Pandian J, Navarro JC. Stroke epidemiology in South, East, and South-East Asia: a review. J Stroke. 2017;19(3):286–94. 10.5853/jos.2017.00234 PMC5647629 29037005

[2] Ng JC, Churojana A, Pongpech S, et al. The current state of acute stroke care in Southeast Asian countries. Interv Neuroradiol. 2019;25(3):291–2963. 10.1177/1591019918811804 PMC6547203 30463501

[3] Sebastian I, Gandhi B, Padmavati N, Sylaja, Paudel R, et al. Stroke systems of care in South-East Asia Region (SEAR): commonalities and diversities. Lancet Reg Health - Southeast Asia. 2023;17:100289. 10.1016/j.lansea.2023.100289 PMC10577144 37849930

[4] Qurat Ul Ain H, Junaid Tahir M, Abbasher Hussien Mohamed Ahmed K, Ahmed F, Mohamed Ibrahim Ali M, Hassan Salih Elhaj E et al. Tele- stroke: a strategy to improve acute stroke care in low- and middle-income countries. Ann Med Surg (Lond). 2024 May 17;86(7):3808–11. Cited 2024 Nov 18. 10.1097/MS9.0000000000002187 PMC11230807 38989217

[5] Demaerschalk BM, Berg J, Chong BW, Gross H, Nystrom K, Adeoye O, et al. Am Telemedicine Association: Telestroke Guidelines Telemedicine e-Health. 2017;23(5):376–89. 10.1089/tmj.2017.0006 PMC5802246 28384077

[6] Dutta D, Kendall J, Holmes C, Murphy P, Black T, Whiting R, et al. Evaluation of a Telephone Advice System for Remote Intravenous Thrombolysis in Ischemic Stroke. Stroke. 2015;46(3):867–9. 10.1161/STROKEAHA.114.008190 25604248

[7] Fong WC, Ismail M, Lo JWT, Li JTC, Wong AHY, Ng YW, et al. Telephone and Teleradiology-Guided Thrombolysis Can Achieve Similar Outcome as Thrombolysis by Neurologist On-site. J Stroke Cerebrovasc Dis. 2015;24(6):1223–8. 10.1016/j.jstrokecerebrovasdis.2015.01.022 25906936

[8] Randles R, Finnegan A. Guidelines for writing a systematic review. Nurse Education Today.June 2023;125 . 10.1016/j.nedt.2023.105803. 36996684

[9] Muengtaweepongsa S, Hunghok W, Harnirattisai T. Poor recognition of prompted treatment seeking even with good knowledge of stroke warning signs contribute to delayed arrival of acute ischemic stroke patients in Thailand. J Stroke Cerebrovasc Dis. 2014;23(5):948–52. 10.1016/j.jstrokecerebrovasdis.2013.07.044 24139409

[10] Naruchorn Kijpaisalratana W, Akharatanawat P, Vongvansikul, Nijasri Suwanela. Outcomes of thrombolytic therapy in acute ischemic stroke: mothership, drip-and-ship, and ship-and-drip paradigms. BMC Neurol. 2020;20:45. 10.1186/s12883-020-1631-9 PMC6998331 32013906

[11] Chutinet A, Keosodsay S, Vorasayan P, Samajarn J, et al. The First 10 Thrombolysis for Acute Ischemic Stroke in Lao People’s Democratic Republic under Teleconsultation from Thailand. J Stroke Cerebrovasc Dis. 2019;28(11):104327. 10.1016/j.jstrokecerebrovasdis.2019.104327 31530479

[12] Sharma S, Padma MV, Bhardwaj A, Sharma A, Sawal N, Thakur S. Telestroke in resource-poor developing country model. Neurol India. 2016;64:934–40. 10.4103/0028-3886.190243 27625232

[13] Vishnu VY, Bhatia R, Khurana D, Ray S, Sharma S, Kulkarni GB, et al. Smartphone-Based Telestroke VsStroke Physician led Acute Stroke Management (SMART INDIA). Ann Indian Acad Neurol. 2022;25(3):422–7. 10.4103/aian.aian_1052_21 PMC9350775 35936578

[14] Tunkl C, Agarwal A, Ramage E, Velez VS, Roushdy T. et.al. Telemedicine networks for acute stroke: An analysis of global coverage, gaps, and opportunities. Int J Stroke.2024:1–13. 10.1177/17474930241298450 PMC11874588 39460528

[15] Arumuganathan P, Adcock AK, Espinosa C, Findley S. Analysis of Telestroke Usage in Rural Critical Access Emergency Departments. Telemed J E Health. 2023 Dec 1 ;29(12):1828–33. Cited 2024 Nov 18. 10.1089/tmj.2022.0408 PMC10714255 37205846

[16] Nardetto L, Dario C, Tonello S, Brunelli MC, Lisiero M, Carraro MG, et al. A one-to-one telestroke network: the first Italian study of a web-based telemedicine system for thrombolysis delivery and patient monitoring. Neurol Sci. 2016;37(5):725–30. 10.1007/s10072-016-2569-y 27032402

[17] Bladin CF, Cadilhac DA. Effect of Telestroke on Emergent Stroke Care and Stroke Outcomes. Stroke. 2014;45(6):1876–80. 10.1161/STROKEAHA.114.003825 24763931

[18] Venketasubramanian N, Yudiarto FL, Tugasworo D. Stroke burden and stroke services in Indonesia. Cerebrovasc Dis Extra. 2022;12(1):53–7. 10.1159/000524161 PMC9149342 35313314

[19] Mazighi M, Meseguer E, Labreuche J, Miroux P, Le Gall C, Roy P, et al. TRUST-tPA trial: Telemedicine for remote collaboration with urgentists for stroke-tPA treatment. J Telemed Telecare. 2017;23(1):174–80. 10.1177/1357633X15615762 26656722

[20] Harvey J, Al Kasab S, Almallouhi E, Guerrero WR, Debenham E, Turner N, et al. Door to needle time and functional outcome for mild ischemic stroke over telestroke. J Telemed Telecare. 2019;25(6):365–9. 10.1177/1357633X18774460 29754560

[21] Sairanen T, Soinila S, Nikkanen M, Rantanen K, Mustanoja S, Färkkilä M, et al. Two years of Finnish Telestroke. Neurology. 2011;76(13):1145–52. 10.1212/WNL.0b013e318212a8d4 21368283

[22] Raulot L, Mione G, Hoffmann CP, Bracard S, Braun M, Brunner A, et al. Intravenous thrombolytic therapy for ischemic stroke via telemedicine compared with bedside treatment in an experienced stroke unit. Eur Res Telemedicine / La Recherche Européenne en Télémédecine. 2015;4(4):119–25.

[23] Mong R, Tiah L, Wong M, Tan C. Improving telestroke treatment times through a quality improvement initiative in a Singapore emergency department. Singap Med J. 2019;60(2):69–74. 10.11622/smedj.2018083 PMC6395833 30009318

[24] Martínez-Sánchez P, Miralles A, Sanz de Barros R, Prefasi D, Sanz- Cuesta BE, Fuentes B, et al. The effect of telestroke systems among neighboring hospitals: more and better? The Madrid Telestroke Project. J Neurol. 2014;261(9):1768–73. 10.1007/s00415-014-7419-3 24957298

[25] Kaminsky AL, Mione G, Omorou Y, Humbertjean L, Bonnerot M, Lacour JC, et al. Outcome of patients with large vessel occlusion stroke after first admission in telestroke spoke versus comprehensive stroke center. J Neurointerv Surg. 2020;12(8):753–7. 10.1136/neurintsurg-2019-015342 31818972

[26] Amalia L. Does the Implementation of a Telestroke Program Play a Role in Improving the Clinical Outcomes of Acute Stroke Patients in South East Asian Region? A Literature Review. Risk Manage Healthc Policy.2025:18 2461–8. 10.2147/RMHP.S517766 PMC12285878 40703311

[27] Chaffin HM, Nakagawa K, Koenig MA. Impact of Statewide Telestroke Network on Acute Stroke Treatment in Hawai’i. Hawaii J Health Soc Welf. 2019;78(9):280–6. PMC6731184 31501825

[28] Agarwal S, Day DJ, Sibson L, Barry PJ, Collas D, Metcalf K et al. Thrombolysis Delivery by a Regional Telestroke Network—Experience From the UK National Health Service. J Am Heart Assoc. 2014;3(1). 10.1161/JAHA.113.000408 PMC3959696 24572251

[29] Lin CH, Lee KW, Chen TC, Lin JH, Liaw KR, Hsiao PJ, et al. Quality and safety of Telemedicine in acute ischemic stroke: Early experience in Taiwan. J Formos Med Assoc. 2022;121(1):314–8. 10.1016/j.jfma.2021.04.024 33994236

[30] Al Kasab S, Harvey JB, Debenham E, Jones DJ, Turner N, Holmstedt CA. Door to Needle Time over Telestroke—A Comprehensive Stroke Center Experience. Telemedicine e-Health. 2018;24(2):111–5. 10.1089/tmj.2017.0067 28753069

